# Supplementary material for: Pharmacological basis of the use of the root bark of Zizyphus nummularia Aubrev. (Rhamnaceae) as anti-inflammatory agent
Source: BMC Complement Altern Med. 2015 Nov 23;15:416. doi: 10.1186/s12906-015-0942-7 (PMC4657250; doi:10.1186/s12906-015-0942-7)
Supplement: Additional file 1: Figure S1. — The effect of octadecahydro-picene-2,3,14,15-tetranone on the viability of RAW 264.7 cells. (PPTX 47 kb) [file 12906_2015_942_MOESM1_ESM.pptx]

Pharmacological basis of the use of the root bark of *Zizyphus nummularia* Aubrev. (*Rhamnaceae*) as anti-inflammatory agent

Sarbani Dey Ray[1, 2], Supratim Ray[3], Muhammad Zia-Ul-Haq[4], Vincenzo De Feo[5,*], Saikat Dewanjee[1,*]

[1]Advanced Pharmacognosy Research Laboratory, Department of Pharmaceutical Technology, Jadavpur University, Kolkata 700032, India.

[2]Dr. B. C. Roy College of Pharmacy & Allied Health Sciences, Bidhannagar, Durgapur 713206, India.

[3]Department of Pharmaceutical Sciences, Assam University, Silchar 788011, India.

[4]Office of Research, Innovation and Commercialization, Lahore College for Women University, Jail Road, Lahore 54000, Pakistan.

[5]Department of Pharmacy, University of Salerno, Fisciano, Salerno 84084, Italy.

* Correspondence: defeo@unisa.it; s.dewanjee@yahoo.com

Abstract

Background: The root bark of *Zizyphus nummularia* (Rhamnaceae) is traditionally used as an anti-inflammatory agent. The current study aimed to explore the anti-inflammatory activity (*in vivo*) of a crude ethanolic extract (EE) and the pure identified octadecahydro-picene-2,3,14,15-tetranone (IC) in the root bark of *Z. nummularia*. IC was further subjected to suitable *in vitro* and *in silico* studies to find out the mechanistic pharmacology.

Methods: EE (100 and 200 mg/kg, p.o.) and (IC) (400 and 600 µg/kg, p.o.) were subjected to *in vivo* anti-inflammatory assays to evaluate the anti-inflammatory activity and predict the probable mechanism(s) of action. Suitable acute (carrageenan-induced paw edema, arachidonic acid-induced ear edema, xylene-induced ear edema) and chronic (cotton pellet granuloma) models were employed to investigate *in vivo* the anti-inflammatory activity. Based on *in vivo* observation, IC was further subjected to *in vitro* assays to estimate the inhibition of nitric oxide (NO), prostaglandin-E_2 (PGE-2) and tumor necrosis factor-α (TNF-α) production in PBS stimulated RAW 264.7 cells. Based on the observation of *in vitro* studies, finally, ADME prediction and molecular docking studies of IC were performed for better understanding of interaction of IC with TNF-α.

Results: Oral administration of EE (100 and 200 mg/kg) exhibited significant inhibition of carrageenan ($p < 0.05$) and arachidonic acid ($p < 0.05$) induced oedema, and the reduced the granuloma tissue formation ($p < 0.05$) in experimental mice. IC (400 and 600 µg/kg, p.o.) exhibited significant ($p < 0.01$) inhibition of carrageenan, xylene and arachidonic acid-induced edema, and reduced the granuloma tissue formation. In *in vitro* assays, IC caused a concentration-dependent inhibition of LPS stimulated NO (up to ~ 67.4% at 50 µM) and TNF-α (~ 84.5% at 50 µM) production. However, the PGE-2 inhibition did not follow dose dependent pattern. Based on *in vitro* observations, the molecular docking has been performed

on the basis of interaction with TNF-α. In *in silico* studies, it was observed that IC showed hydrogen bonding with GLN 47 amino acid residue of TNF-α protein.

Conclusions: IC possibly produces anti-inflammatory activity through inhibition of TNF-α and NO production.

Keywords: *Zizyphus nummularia*, octadecahydro-picene-2,3,14,15-tetranone, NO, TNF-α, molecular docking

Background

Inflammation is the complex biological response to pathophysiological events mediated by various signalling molecules produced by leukocytes, macrophages and mast cells [1]. During inflammation, enhanced vascular permeability coupled with migration of blood corpuscles into the inflammatory site/s causes oedema, erythema and pain. Various inflammatory mediators, namely nitric oxide (NO), prostaglandin-2 (PGE-2), interleukins (ILs) and tumor necrosis factor (TNF-α) play an imperative role during the progress of inflammation [2]. Inflammation is major cause of morbidity throughout the world [3]. If untreated, it may lead to various associated diseases like arthritis, atherosclerosis, and even cancer [4-6]. Serious adverse effects of most of commercially available anti-inflammatory drugs largely encourage the development of new, target specific and less toxic anti-inflammatory agents from plants [7]. Many Indian herbs have been claimed to exert notable anti-inflammatory activity without producing considerable untoward effects [8-9]. Recently computer-aided drug design by *in silico* computer aided drug design is being employed in rational drug discovery to understand the inhibitor-receptor interactions and predict the inhibitory activity of new compounds. Therefore, the combination of ethnopharmacological literature and modern scientific tools including molecular docking is now believed to offer a holistic approach of novel drug discovery.

Zizyphus nummularia Aubrev. (*Rhamnaceae*), a thorny small bush or shrub, grows in abundance in the grazing lands of the arid and semi-arid regions of India. The plant is used in traditional medicine as analgesic, anti-inflammatory, antitussive, anthelmintic, and anti-cancer drug [10-12]. Leaves and root bark of the plant are used as the remedy of inflammation by the local communities of eastern India. While, much work exists on extracts of leaves in animal model proving the anti-inflammatory action [13-16]. On other hand, there is no literature on rationalization of anti-inflammatory activity of root bark of *Z. nummularia*

by experimental models. However, the root barks of different species under the same genus, *Zizyphus*, have been reported to possess significant anti-inflammatory activity [17-19]. Considering the ethnopharmacological relevance and the existing literatures in support of anti-inflammatory activities of the root bark of *Zizyphus* species, the present study was designed to rationalize the anti-inflammatory activity of the crude extract and of the isolated compound, octadecahydro-picene-2,3,14,15-tetranone, from the root bark of *Z. nummularia* employing *in vivo* animal models. The earlier reports regarding anti-inflammatory activity of some small molecules bearing similar pentacyclic structure [20-22] encouraged us to pursue the study of anti-inflammatory activity of this isolated compound. Based on the observation of the *in vivo* bioassay, the mechanism of action of the isolated compound was studied with respect to *in vitro* assays in murine monocytic macrophage cell line (RAW 264.7). Therefore, IC was further subjected to *in silico* study to predict its possible orientation at receptor level.

Materials and methods

Test materials

Root bark of *Z. nummularia* was collected in September 2010 from Durgapur, India and authenticated (Ref. CNH/I-I/20/2010/Tech.II/171) by Dr. V. P. Parsad, Taxonomist, Central National Herbarium, Botanical Survey of India, Shibpur, India. A voucher specimen (BCRCP/DP/PT/02/06) was deposited at Dr. B. C. Roy College of Pharmacy & Allied Health Sciences, Durgapur, India for future reference. The detailed methods of extraction, isolation and structure elucidation have been described in our previous publication [12]. The structure of IC has been depicted in Figure 1. EE and IC were suspended in Tween-80 (1%) prior to each animal experiment. For *in vitro* assays, IC was solubilised in DMSO in a master plate (resultant $\leq$ 0.4% DMSO in contact to cells to avoid DMSO induced cytotoxicity). Briefly, the IC solution of different concentrations in 100 % DMSO in a master plate was diluted (1 in

25 dilution resulting 4 % DMSO) in a drug dilution plate. Finally, IC solution of desired concentrations was introduced into cells (1 in 10 dilution resulting 0.4 % DMSO).

Chemicals

Cell line RAW264.7 was obtained from SIGMA-RBI, Switzerland. Dulbecco's Modified Eagle Medium (DMEM), phosphate buffered saline (PBS) and Griess reagent were procured from Invitrogen, Carlsbad, USA. Lipopolysaccharide (LPS), foetal bovine serum (FBS) from *E. coli* (serotype 0111:B4), dimethylsulfoxide (DMSO) and sodium nitrite were procured from Sigma (St Louis, USA). All solvents and reagents used were of analytical grade.

***In vivo* anti-inflammatory activity**

Swiss albino mice (♂, 25 ± 5 g, age: 2-3 months) were housed in standard polyprophylene cages (3 mice/cage) under standard laboratory conditions of 12:12 light-dark cycle, temperature (20 ± 2 ºC), relative humidity (55 ± 5%), standard diet (Hindustan Liver Ltd. Mumbai, India) and water *ad libitum*. The animal experiments were conducted in accordance with the institutional animal ethical committee of Dr. B. C. Roy College of Pharmacy and AHS (Reg No: BCRCP/IAEC/8/2012).

Carrageenan-induced paw oedema

Mice were divided into six groups (n = 6) and the acute inflammation was induced by carrageenan [23]. Pedal inflammation was induced by a single subcutaneous injection of carrageenan (0.1 ml, 1%, w/v, in normal saline) into the right paw of each mouse under the subplantar aponeurosis. Thirty minutes prior to carrageenan injection, the first group of mice received orally tween-80 (1%) and served as inflammation control. The second and third group were treated orally with EE at the doses of 100 and 200 mg/kg, respectively, while, fourth and fifth groups were treated with IC at the doses of 400 and 600 µg/kg, respectively. A group of mice was treated with the standard anti-inflammatory drug, aspirin (positive control). The paw volume was measured by dipping the foot in the mercury bath of a

plethysmometer up to the anatomical hairline on lateral malleolus and compared with control animals. The paw volumes were recorded at 0, 1, 2, 3, 4 and 5 h following carrageenan injection.

Arachidonic acid and xylene-induced right ear oedema

Inflammation was induced in mice (n = 6/group) by applying on the inner and outer surfaces of the right ear 30 µl of following irritants: arachidonic acid 0.1 mg/µl in acetone [24] and xylene [25]. Thirty minutes prior to irritants' treatment, two groups of mice received orally tween-80 (1%) and served as control group. The other groups were treated orally with EE (100 and 200 mg/kg) and IC (400 and 600 µg/kg), respectively. Two groups of mice were treated with the standard anti-inflammatory, aspirin (10 mg/kg) (positive control). The left ear served as normal control. Thirty minutes after the arachidonic acid injection, the mice under different groups were subjected to CO_2 euthanasia and sacrificed by cervical dislocation. Both ears were removed and weighed. The data were represented as percent of oedema weight.

Cotton pellet-induced granuloma

This study was carried out following the protocol of Ismail and co-authors [27] with little modification. Sterile cotton pellets (10 ± 0.5 mg) were implanted subcutaneously on the backs of mice. The six groups (n = 6/group) of mice were treated with aqueous tween 80 (1%), EE (100 and 200 mg/kg), IC (400 and 600 µg/kg) and aspirin (10 mg/kg) orally, once daily over 7 consecutive days. On day 8, the mice were subjected to CO_2 euthanasia and sacrificed by cervical dislocation. The cotton pellet were removed, dried overnight at 60 ºC and weighed.

***In vitro* anti-inflammatory activity of IC**

Cell culture

The RAW 264.7 cells were maintained in DMEM supplemented with FBS (10%), glucose (4.5 g/l), sodium pyruvate (1 mM), L-glutamine (2 mM), streptomycin (50 μg/ml) and penicillin (50 U/ml) at 37 °C and 5% CO_2. The medium was routinely changed on alternate days. The cells were passaged by trypsinization (Trypsin-EDTA) to disrupt cell monolayer at confluence while splitting RAW264.7 cells for the routine culture and plating the cells for the *in vitro* assays.

Cytotoxicity assay

The cytotoxic effect of IC was determined by cell viability assay. Briefly, Cell suspension was seeded into a 96-well plate (~ 4×10^5 cells/well) and incubated for 12 h (37 °C; 5 % CO_2) to allow cell attachment. The cells were then incubated with IC (1-1000 μM) at 37 ºC and 5% CO_2 tension. The cell viability was assessed at 2 h and 4 h by MTT assay [28]. IC did not cause any loss of cell viability up to 100 μM as compared with untreated RAW264.7 cells (data were shown in Supple figure 1).

Estimation of NO, PGE-2 and TNF-α inhibitory activity

Cell suspension was seeded into a 96-well plate (~ 4×10^5 cells/well) and incubated for 12 h (37 °C; 5 % CO_2) to allow cell attachment. The cells were then stimulated with lipopolysaccharide (1 μg/ml) and different concentrations of IC. Nitrite accumulation, an indicator of NO synthesis, was measured in culture media based on a diazotization reaction using the Griess reagent [29]. The nitrite concentration was measured using sodium nitrite as a standard. PGE-2 and TNF-α in the supernatant were measured using ELISA kits (eBioscience, USA) according to manufacturer's instructions.

***In silico* ADME prediction and molecular docking studies of IC**

The pharmacokinetic profile of IC was assessed using absorption, distribution, metabolism, elimination (ADME) prediction methods. The compound was subjected to evaluation by the QikProp® (Version 3.2) module of the Maestro Schrodinger (MS) software for prediction of

pharmacokinetic properties. IC was neutralized before being subjected to QikProp® analysis and significant pharmacokinetic properties consisting of principal descriptors such as mol_MW, SASA, FOSA, FISA, PISA, volume, donarHB, accptHB, QPlogPo/w, human oral absorption, percent human oral absorption, #rtvFG, CNS activity and finally Lipinski's rule of five. The details of QikProp® properties and descriptors are listed in Table 1. The compliance of the IC to the Lipinski's rule of five holds the potential for the molecule to be further developed in drug design programmes.

Active site prediction and molecular docking

Preparation of the ligand

TNF-α inhibitory activity of IC in the *in vitro* assay was taken in to consideration to study the mode of inhibition of the selected TNF-α protein. The 3D structures of the IC was built using Maestro 9.0 build panel and prepared by LigPrep 2.3 version v23118 (Schrödinger, LLC., USA). The application uses Optimized Potentials for Liquid Simulations (OPLS) 2005 force field and energy minimized with Macromodel-v97110.

Preparation of the protein and prediction of active site

A docking study was carried out at the receptor site of TNF-α protein to find out the putative binding mode of the isolated compounds. The crystal structure of recombinant human TNF-α with a resolution of 2.30 Å was retrieved from the protein data bank (PDB ID: 1A8M) [30]. The structure was prepared by the protein preparation wizard within the Maestro Schrödinger®9.0 module, which was further utilized to predict the possible active site. As the selected TNF-α protein was devoid of associated co-crystallized ligand, therefore the location of the primary binding site on a receptor was unknown. Therefore, the Sitemap® (version 2.3, Schrödinger, LLC, New York, NY, 2009) module in Maestro Schrödinger® 9.0 version v23118 was utilized to detect the possible potential binding cavities within the receptor. The outcome of sitemap using OPLS 2005 force field resulted in the detection of two binding

sites [31] and the highest scored (Table 2) binding site (Figures 2 and 3) was selected for the molecular docking.

Statistical analysis

The experimental data were statistically analyzed by the One-way Analysis of Variance (ANOVA) and expressed as mean ± S.E.M. followed by Dunnett's t-test using computerised GraphPad InStat version 3.05, Graph pad software, USA. The differences are considered significant when $p < 0.05$.

Results and Discussion

In vivo studies to validate the effect of EE and IC

Effects on carrageenan-induced right paw edema

Carrageenan-induced inflammation model is a well-established method used for acute inflammation. The edema expansion follows biphasic responses. The early phase (within 1 h) is mediated by the discharge of autacoids, viz. histamine, kinins and serotonin, while the later phase (after 1 h) involves prostaglandins; the link between the two phases is provided by kinins [32]. Oral administration of EE (100 and 200 mg/kg) exhibited significant inhibition of carrageenan-induced inflammation (Figure 4A). The EE (100 and 200 mg/kg) exhibited significant ($p < 0.01$) anti-inflammatory activity 3 h after carrageenan injection. On other hand, IC (400 and 600 µg/kg) treatment significantly ($p < 0.05$-0.01) inhibited carrageenan induced paw edema 2 h after carrageenan administration. The maximum inhibitory values of oedema at 3 h post-carrageenan were 16.2 and 17.9% with the doses of 100 and 200 mg/kg of EE, respectively. However, IC (400 and 600 µg/kg) ensured maximum inhibition of 32.0 and 35.2%, respectively, after 4 h of carrageenan injection. The anti-inflammatory effects of EE and IC were compared with the standard drug, aspirin (10 mg/kg), which exhibited significant inhibition of paw edema 1 h onward post-carrageenan treatment. Based on this

observation, this activity might be attributed to the inhibition of the release of aforementioned inflammatory mediators.

Effect on arachidonic acid and xylene-induced right ear oedema

The arachidonic acid and xylene can cause an acute inflammatory response and lead to severe vasodilation and oedematous changes [33]. The results showed that EE (200 mg/kg) exhibited significant ($p < 0.05$) suppression of arachidonic acid and xylene-induced ear oedema in mice, while the effect of IC (400 and 600 µg/kg) is more pronounced ($p < 0.01$) (Figure 4B). The activity of IC (600 µg/kg) was found nearly comparable to that of positive control aspirin (10 mg/kg). Arachidonic acid is a precursor of PGE-2. Besides, arachidonic acid also can act as a second messenger to regulate many cellular processes including nitric oxide formation [34]. Therefore, the observed effect may be due to inhibition of PGE-2 and/or NO production. On other hand, xylene causes the release of pro-inflammatory mediators from sensory neurons that act on peripheral target cells such as mast cells and other immune cells producing neurogenic inflammation [35].

Effect on granuloma tissue formation

The inflammatory granuloma tissue formation is a feature of chronic inflammation. It evaluates the effects on macrophage dysfunction and granuloma formation [3]. Macrophage activation during the process of chronic inflammation causes release of pro-inflammatory mediators, including TNF-α, and participates in the subsequent process of inflammation [36]. Figure 4C depicted that the effect of EE and IC on granuloma tissue formation. EE exhibited significant ($p < 0.05$) inhibition of dry weight of the cotton-pellet granuloma. The inhibitory values for 100, 200 mg/kg of the EE were ~ 30.3 and 33.4%, respectively. IC exhibited significant ($p < 0.01$) inhibition of granuloma tissue formation with inhibition values of 40.2 and 42.1% for the doses of 400 and 600 µg/kg, respectively. The effect of IC (600 µg/kg) was found nearly comparable to that of positive control, the standard anti-inflammatory agent,

aspirin (10 mg/kg), which exhibited an inhibitory value of ~ 43.3%. The inhibitory effect of test materials may be due to inhibition of macrophage activation. Based on the results of *in vivo* studies, IC was further subjected to *in vitro* experiment to elucidate its possible mechanism.

In vitro studies to predict the probable mechanism

Effect of IC on NO, PGE-2 and TNF-α production

The effects of IC on LPS stimulated NO, PGE-2 and TNF-α production in RAW 264.7 cells were depicted in Figure 5. LPS-induced macrophage activation increased the production of pro-inflammatory cytokines and inflammatory mediators, including NO, PGE-2 and TNF-α [37]. IC caused a concentration-dependent inhibition of LPS-stimulated NO production up to ~ 67.4% at the highest used dose of 50 µM. NO plays an important role in various inflammatory conditions and in tissues is susceptible to manipulation by pro-inflammatory cytokines [38]. The inhibition of TNF-α production also follows a concentration-dependent manner. IC caused a maximum inhibition of LPS stimulated TNF-α production of ~84.5% at the dose of 50 µM and the steady state inhibition (> 80%) attained between 1-50 µM concentration range. However, the PGE-2 inhibition did not follow a dose-dependent pattern. The maximum inhibition of ~61.4% was observed between the 1-5 µM. Based on *in vitro* observations, it would be hypothesized that IC possibly acts through inhibition of TNF-α production. Therefore, molecular docking has been performed on the basis of interaction with TNF-α.

In silico observations

ADME

The predicted value of Lipinski's rule of five for IC is within the range of stipulated values. The predicted values of each individual parameters necessary for Lipinski's rule of five (mol_MW, QPlogPo/w, donorHB and accptHB) are also well within the recommended range

(Table 3) which indicate that IC has property of drug-likeness [39]. The calculated values of total solvent accessible surface area (SASA) along with its hydrophobic component (FOSA), hydrophilic component (FISA), the π component (PISA) and volume of IC are within the stipulated ranges which favours the fixation of IC with hydrophilic-hydrophobic contour of TNF-α receptor. The predicted qualitative human oral absorption of IC is high (3 in our case) and the percent of human oral absorption value is 77.61%, very close to the recommended values. The number of reactive functional groups that can produce reactivity and toxicity problems *in vivo* is zero. Finally, the predictive central nervous activity of IC is –2, which indicates that IC is CNS inactive.

Active site prediction and molecular docking

In the present assessment of ligand-receptor interactions using Glide, IC showed hydrogen bonding with GLN 47 amino acid residue (Figure 6). The G score of -3.286 obtained by the dock pose of IC complemented by the hydrophilic-hydrophobic contour of TNF-α protein (Figure 7). The hydrogen bond interaction occurred with oxygen atom of the isolated compounds and GLN 47. The oxygen atom served as hydrogen bond acceptor with the $–NH_2$ group of glutamine. This also correlates with the calculated values of accptHB of IC (5.5 in our case). Molecular docking study also reveals the best possible environment necessary for drug receptor interaction. The hydrophilic-hydrophobic domain of the predicted active site of TNF-α comprises of ARG 131, ASP 45, LYS 90, GLN 47, ASN 46, GLU 135, GLN 27, GLN 25, LEU 26, GLU 23 and GLY 24 amino acid residues (Figure 8). The similar types of molecular docking were reported earlier to understand the probable interactions between TNF-α protein and IC [40,41].

In the present study, we found that the root bark of *Z. nummularia* exhibited significant anti-inflammatory activity. We also observed that, octadecahydro-picene-2,3,14,15-tetranone isolated from the root bark of *Z. nummularia* exhibited significant anti-inflammatory activity.

To predict the mechanism, *in vitro* assays were performed to see the effect of IC on LPS stimulated NO, PGE-2 and TNF-α production in RAW 264.7 cells. Among the tested mediators, IC significantly inhibited LPS stimulated TNF-α and NO production. However, the inhibitory effect on TNF-α production has been found more pronounced. Based on quantitative value/inhibition characteristics, molecular docking studies were performed on TNF-α protein. Molecular docking study further helped in supporting the observed TNF-α selectivity. Based on these observation, IC can be regarded as an anti-inflammatory agent with possible inhibitory effect on NO and TNF-α production. Therefore, the compound may have clinical potential for the treatment of inflammation in future.

Acknowledgements

The authors wish to thank the staffs of Division of Pharmaceuticals & Fine chemicals, Department of Chemical Technology, University of Calcutta for providing computational support to carry out in silico experiment. One of the authors (SDR) also wishes to acknowledge the authority of Dr B C Roy College of Pharmacy & A.H.S for providing the necessary facility for carrying out the animal experiment. Authors are thankful to All Indian Council for Technical Education, New Delhi, India for providing financial support (Ref. No. RID/PRES.-2009-10/RPS).

Authors' contribution

SDR participated in phytochemical analysis and pharmacological assays. SR performed *in silico* studies. SD designed and supervised the experiment. MZUH and VDF put important views to during designing of experiment. SD, VDF and MZUH equally contributed in writing this manuscript. All authors read and approved the final manuscript.

Conflicts of Interest

The authors declare that there is no conflict of interest.

Supplementary file

Supplementary file showed the cytotoxic effect of octadecahydro-picene-2,3,14,15-tetranone.

References

[1] Sengar N, Joshi A, Prasad SK, Hemalatha S. Anti-inflammatory, analgesic and anti-pyretic activities of standardized root extract of *Jasminum sambac*. J Ethnopharmacol. 2015;160:140-8.

[2] Dewanjee S, Dua TK, Sahu R. Potential anti-inflammatory effect of *Leea macrophylla* Roxb. leaves: A wild edible plant. Food Chem Toxicol. 2013;59:514-20.

[3] Dewanjee S, Maiti A, Sahu R, Dua TK, Mandal SC. Study of anti-inflammatory and antinociceptive activity of hydroalcoholic extract of *Schima wallichii* bark. Pharm Biol. 2009;47:402-7.

[4] Schett G. Rheumatoid arthritis: inflammation and bone loss. Wiener Medizinische Wochenschrift. 2006;56:34-41.

[5] Libby P, Ridker P, Maseri A. Inflammation and Atherosclerosis. Circulation 2002;105:1135-43.

[6] Karin M, Greten FR. NF-kappa B: linking inflammation and immunity to cancer development and progression. Nat Rev Immunol. 2005;5:749-59.

[7] Dewanjee S, Mandal V, Sahu R, Dua TK, Manna A, Mandal SC. Anti-inflammatory activity of a polyphenolic enriched extract of *Schima wallichii* bark. Nat Prod Res. 2011;25:696-703.

[8] Chopra RN, Nayar SL, Chopra IC. Glossary of Indian medicinal plants, Council of Scientific & Industrial Research, New Delhi, India, 1956.

[9] Khanra R, Dewanjee S, Dua TK, Sahu R, Gangopadhyay M, De Feo V, et al. *Abroma augusta* L. (Malvaceae) leaf extract attenuates diabetes induced nephropathy and

cardiomyopathy via inhibition of oxidative stress and inflammatory response. J Transl Med. 2015;13:6.

[10] Shah AH, Tariq M, Al-Yahya MA. Studies on the alkaloidal fraction from the stem bark of *Zizyphus nummularia*. Fitoterapia. 1990;61:46-9.

[11] Bachaya AH, Iqbal Z, Khan MN, Sindhu Z, Jabbar A. Anthelmintic activity of *Ziziphus nummularia* (bark) and *Acacia nilotica* (fruit) against Trichostrongylid nematodes of sheep. J Ethnopharmacol. 2009;123:325-9.

[12] DeyRay S, Dewanjee S. Isolation of a new triterpene derivative and in vitro and in vivo anticancer activity of ethanolic extract from root bark of *Zizyphus nummularia* Aubrev. Nat Prod Res. 2014;DOI:10.1080/14786419.2014.983921.

[13] Kumar S, Garg VK, Sharma PK: A review of *Ziziphus nummularia.* Pharmacologyonline. 2010;2:565-74.

[14] Goyal M, Ghosh M, Nagori BP, Sasmal D. Analgesic and anti-inflammatory studies of cyclopeptide alkaloid fraction of leaves of *Ziziyphus nummularia.* Saudi J Biol Sci. 2013;20:365-71.

[15] Soliman YH: Topical Anti-inflammatory and wound healing activities of herbal gel of *Ziziphus nummularia* L. (F. *Rhamnaceae*) leaf extract. Int J Pharm. 2011;7:862-7.

[16] Goyal M, Sasmal D, Nagori BP: Analgesic and anti-inflammatory activity of ethanolic extract of *Zizyphus nummularia*. Res J Med Plant .2012;6:521-8.

[17] Yadav A, Singh P. Analgesic and anti-inflammatory activities of *Zizyphus rugosa* root barks. J Chem Pharm Res. 2010;2:255-9.

[18] Adzu B, Amos S, Wambebe C, Gamaniel K. Anti-nociceptive activity of the aqueous extract of *Ziziphus spinachristi* root bark. Fitoterapia. 2001;72:344-50.

[19] Borgi W, Recio M-C, Rios JL, Chouchane N. Anti-inflammatory and analgesic activities of flavonoid and saponin fractions from *Zizyphus lotus* (L.) Lam. S Afr J Bot. 2008;74:320-4.

[20] Yuan G, Wahlqvist ML, He G, Yang M, MD DL. Natural products and anti-inflammatory activity. Asia Pac J Clin Nutr. 2006;15:143-52

[21] Safayhi H, Sailer ER. Anti-inflammatory actions of pentacyclic triterpenes. Planta Med. 1997;63:487-93.

[22] Patočka J. Biologically active pentacyclic triterpenes and their current medicine signification. J Appl Biomed. 2003;1:7-12.

[23] Sofidiya MO, Odukoya OA, Adedapo AA, Mbagwu HOC, Afolayan AJ, Familoni OB. Investigation of the anti-inflammatory and antinociceptive activities of *Hymenocardia acida* Tul. (Hymenocardiaceae). Afr J Biotechnol. 2010;9:8454-9.

[24] Young JM, Spires DA, Bedord CJ, Wagner B, Ballaron SJ, De Young LM. The mouse ear inflammatory response to topical arachidonic acid. J Invest Dermatol. 1984;82:367-71.

[25] Nunez Guillen ME, Emim JA, Souccar C, Lapa AJ. Analgesic and anti-inflammatory activities of the aqueous extract of *Plantago major* L. Int J Pharmacogn.1997;35:99-104.

[27] Ismail TS, Gapalakrisan S, Begum VH, Elango V. Anti-inflammatory activity of *Salacia oblonga* Wall. and *Azima tetracantha* Lam. J Ethnopharmacol. 1997;56:145-52.

[28] Dua TK, Dewanjee S, Gangopadhyay M, Khanra R, Zia-Ul-Haq M, De Feo V. Ameliorative effect of water spinach, *Ipomea aquatica* (Convolvulaceae), against experimentally induced arsenic toxicity. J Transl Med. 2015;13:81.

[29] Titheradge MA. The enzymatic measurement of nitrate and nitrite, Methods Mol Biol. 1998;100:83-91.

[30] Reed C, Fu ZQ, Wu J, Xue YN, Harrison RW, Chen MJ, et al. Crystal structure of TNF-alpha mutant R31D with greater affinity for receptor R1 compared with R2. Protein Eng. 1997;10:1101-7.

[31] Halgren T. New method for fast and accurate binding-site identification and analysis. Chem Biol Drug Des. 2007;69:146-8.

[32] Mothana RAA. Anti-inflammatory, antinociceptive and antioxidant activities of the endemic Soqotraen *Boswellia elongata* Balf. F. and *Jatropha unicostata* Balf. F. in different experimental models. Food Chem Toxicol. 2011;49:2594-9.

[33] Kim HD, Cho HR, Moon SB, Shin HD, Yang KJ, Park BR, et al. Effects of β-glucan from *Aureobasidium pullulans* on acute inflammation in mice. Arch Pharm Res. 2007;30:323-8.

[34] Signorello MG, Segantin A, Leoncini G. The arachidonic acid effect on platelet nitric oxide level. Biochem Biophys Acta. 2009;1791:1084-92.

[35] Richardson JD, Vasko MR. Cellular mechanisms of neurogenic inflammation. J Pharmacol Exp Ther. 2002;302:839-45.

[36] Nair V, Singh S, Gupta YK. Anti-granuloma activity of *Coriandrum sativum* in experimental models. J Ayurveda Integr Med. 2013;4:13-8.

[37] Song M, Park H-J. Anti-inflammatory effect of *Phellinus linteus* grown on germinated brown rice on dextran sodium sulfate-induced acute colitis in mice and LPS-activated macrophages. J Ethnopharmacol. 2014;154:311-8.

[38] Bucci M, Roviezzo F, Posadas I, Yu J, Parente L, Sessa WC, et al. Endothelial nitric oxide synthase activation is critical for vascular leakage during acute inflammation *in vivo*. Proc Natl Acad Sci USA. 2005;102:904.

[39] Lipinski CA, Lombardo F, Dominy BW, Feeney PJ. Experimental and computational approaches to estimate solubility and permeability in drug discovery and development settings, Adv Drug Deliv Rev. 2001;46:3-26.

[40] Haider S, Alam MS, Hamid H, Shafi S, Nargotra A, Mahajan P, et al. Synthesis of novel 1,2,3-triazole based benzoxazolinones: their TNF-α based molecular docking with in-vivo anti-inflammatory, antinociceptive activities and ulcerogenic risk evaluation. Eur J Med Chem. 2013;70 :579-88.

[41] Mouchlis VD, Melagraki G, Mavromoustakos T, Kollias G, Afantitis A. Molecular modeling on pyrimidine-urea inhibitors of TNF-α production: an integrated approach using a combination of molecular docking, classification techniques, and 3D-QSAR CoMSIA. J Chem Inf Model. 2012;52:711-23.

Table 1. The Qikprop® properties and descriptors.

Sl no.	Descriptor	Description	Recommended range
1	mol_MW	Molecular weight of the molecule	130.0-725.0
2	SASA	Total solvent accessible surface area (SASA) in square angstroms using a probe with a 1.4 A^0 radius	300.0-1000.0
3	FOSA	Hydrophobic component of the SASA (saturated carbon and attached hydrogen)	0.0-750.0
4	FISA	Hydrophilic component of the SASA (SASA on N, O and hydrogen on heteroatom)	7.0-330.0
5	PISA	Π (carbon and attached hydrogen) component of SASA	0.0-450.0
6	volume	Total solvent-accessible volume in cubic angstroms using a probe with 1.4 A^0 radius	500.0-2000.0
7	donorHB	Estimated number of hydrogen bonds that would be donated by the solute to water molecules in an aqueous solution. Values are averages taken over a number of configurations, so they can be non-integer	0.0-6.0
8	accptHB	Estimated number of hydrogen bonds that would be accepted by the solute to water molecules in an aqueous solution. Values are averages taken over a number of configurations, so they can be non-integer	2.0-20.0
9	QPlogP o/w	Predicted octanol / water partition coefficient	-2.0-6.5
10	Human oral absorption	Predictive qualitative human oral absorption. The assessment uses a knowledge-based set of rules, including checking for suitable values percent human oral absorption, number of metabolites, number of rotatable bonds logP, solubility and cell permeability	1, 2, 3 for low, medium and high absorption respectively
11	% human oral absorption	It predicts human oral absorption on 0 to 100% scale. The prediction is based on a quantitative multiple linear regression model. This property usually correlates well with human oral absorption.	>80% is high <25% is poor
12	#rtvFG	This particular descriptor indicates the number of reactive functional groups. The presence of these groups can lead to decomposition, reactivity, or toxicity problems *in vivo*.	0 to 2.0
13	CNS	Predictive central nervous activity on a –2 (inactive) to +2 (active) scale.	-2.0 to 2.0
14	Lipinski's rule of five	Lipinski's rules of five are: mol_MW< 500, QPlogPo/w < 5, donorHB ≤ 5, accptHB ≤ 10. Compounds that satisfy these rules are considered drug like. (The "five" refers to the limits, which are multiples of 5).	Maximum is 4

Table 2. Top-ranked SiteMap® prediction for receptor binding sites.

Sl. no	Title	Site score
1	Sitemap_site1	0.643
2	Sitemap_site2	0.566

Table 3. Pharmacokinetic prediction of selected compound (IC) by QikProp® 3.2.

Sl no.	Descriptor	Predicted values of IC
1	mol_MW	356.46
2	SASA	603.7
3	FOSA	326.13
4	FISA	209.07
5	PISA	68.5
6	volume	1115.35
7	donorHB	2
8	accptHB	5.5
9	QPlogPo/w	2.498
10	Human oral absorption	3
11	% human oral absorption	77.61
12	#rtvFG	0
13	CNS	-2.0
14	Lipinski's rule of five	0

Figure captions

Figure 1. The structure of isolated picene compound, octadecahydro-picene-2,3,14,15-tetraone.

Figure 2. The centroid of the sitemap_site1 used in the generation of grid of TNF-α receptor. Hydrophobic map: yellow mesh; hydrogen bond (HB) donor map: blue mesh; HB acceptor map: red mesh. White points indicate generated site points.

Figure 3. The centroid of the sitemap_site1 within the TNF-α receptor site.

Figure 4. Effect of EE and IC in acute and chronic inflammations in mice. Panel A: Effect on carrageenan induced paw edema in mice. Panel B: Effect on xylene and arachidonic acid-induced mouse ear model. Percent of oedema weight = 100 x $(W_R-W_L)/W_L$; where W_R is the mass of right ear, W_L mass left ear. Panel C: Effect on cotton pellet granumola in experimental mice. Values are expressed as mean ± SE (n = 6). *$p < 0.05$ compared with control group. **$p < 0.01$ compared with control group.

Figure 5. Effect of IC in NO, PGE-2 and TNF-α production in PBS stimulated RAW 264.7 cells. Values are expressed as mean ± SE (n = 3).

Figure 6. 3D view of docking pose of minimum energy structure complex of IC docked at the predicted active site of TNF-α (PDB ID: 1A8M) viewed using Glide XP visualizer of Schrödinger Maestro. Hydrogen bond is shown as yellow dash and bonded with GLN 47.

Figure 7. Hydrophilic-lipophilic contour (White portion include Hydrophilic domain of TNF-α and brown portion indicate Hydrophobic domain of TNF-α).

Figure 8. The key amino acids were shown within the active site of TNF-α after docking.

Supple figure 1. The effect of octadecahydro-picene-2,3,14,15-tetranone on the viability of RAW 264.7 cells.

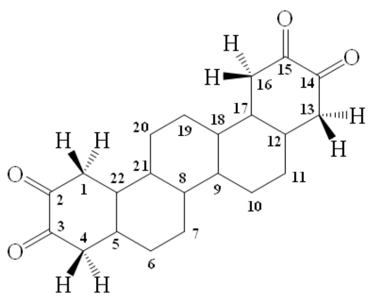

Fig. 1

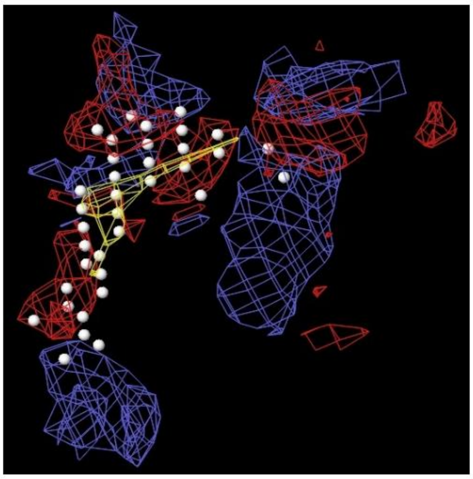

Fig. 2

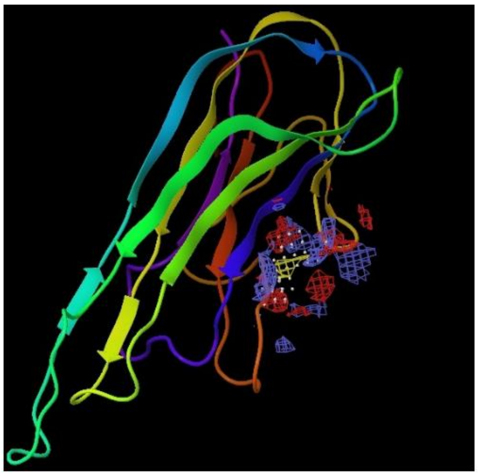

Fig. 3

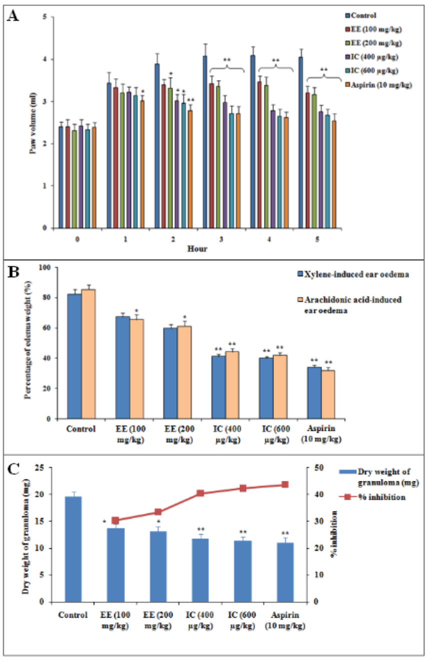
A
Paw volume (ml)
Hour
Control
EE (100 mg/kg)
EE (200 mg/kg)
IC (400 µg/kg)
IC (600 µg/kg)
Aspirin (10 mg/kg)
B
Percentage of edema weight (%)
Xylene-induced ear oedema
Arachidonic acid-induced ear oedema
Control
EE (100 mg/kg)
EE (200 mg/kg)
IC (400 µg/kg)
IC (600 µg/kg)
Aspirin (10 mg/kg)
C
Dry weight of granuloma (mg)
% inhibition
Dry weight of granuloma (mg)
% inhibition
Control
EE (100 mg/kg)
EE (200 mg/kg)
IC (400 µg/kg)
IC (600 µg/kg)
Aspirin (10 mg/kg)

Fig. 4

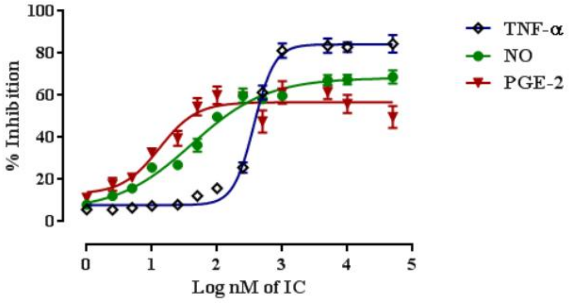

Fig. 5

559

560

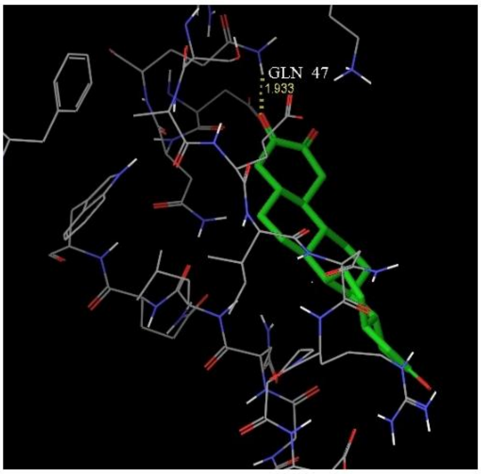

Fig. 6

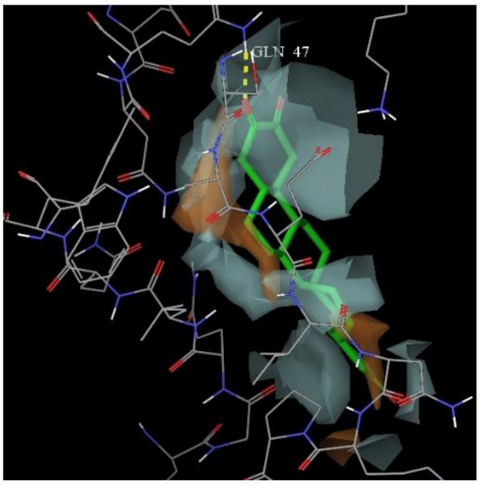

Fig. 7

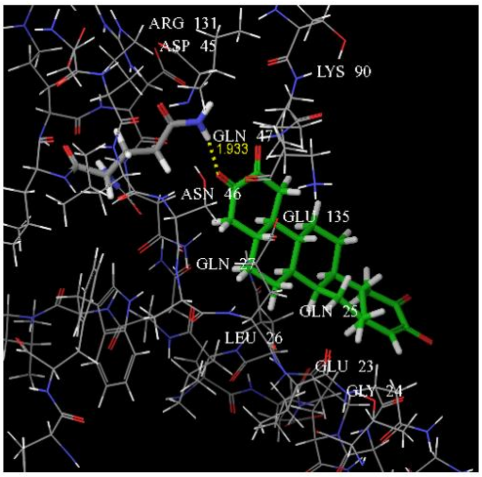
ARG 131
ASP 45
LYS 90
GLN 47
1.933
ASN 46
GLU 135
GLN 27
GLN 25
LEU 26
GLU 23
GLY 24

Fig. 8

565
